# Book Reviews

**Published:** 1904-08

**Authors:** 


### BOOK REVIEWS.

---

INTERNATIONAL CLINICS. A Quarterly of Illustrated Clinical Lectures and especially prepared original articles on Treatment, Medicine, Surgery, Neurology, Pediatrics, Obstetrics, Gynecology, Orthopedics, Pathology, Dermatology, Ophthalmology, Otology, Rhinology, Laryngology, Hygiene and other topics of interest to students and practitioners. By leading members of the medical profession throughout the world. Edited by A. O. J. KELLY, A.M., M.D., Philadelphia. Volume I. Fourteenth series. 1904. Philadelphia: J. B. Lippincott Company. (Cloth, $2.00.)

The first volume of the fourteenth series of the clinics presents an admirable appearance, even outstripping most of its predecessors,—which is saying a good deal. It is, however, no exaggeration to affirm that in material, illustration, and general good looks this is a surprising volume,—one that reflects the energy of its publishers and the ability of its contributors.

Under the head of treatment are five contributions, as follows: the chlorid reduction treatment of parenchymatous nephritis, by F. Widal and A. Javal; adonidin, a clinical study, by Reynold W. Wilcox; therapeutic applications of colloid silver, by Netter and Solomon; what is the cure for neurasthenia, by Robert T. Edes; treatment of gastric and allied conditions, by George W. McCaskey. In the section on medicine are four articles by N. S. Davis, Henry W. Cattell, Henry B. Favill, and James J. Walsh respectively; while in the department of surgery are articles by Carl Beck, Clark and Luther, J. McFadden Gaston, Charles P. Noble, and Frederick Griffith. The articles on gynecology are contributed by Francis H. Davenport and Daniel H. Craig; and on neurology, William Broaddus Pritchard contributes the sole lecture, which is on peripheral neuritis. The progress of medicine during 1903 is reported under the heads of medicine, surgery, and treatment by David L. Edsall, Joseph C. Bloodgood, and A. A. Stevens.

It would be difficult to group better literature by abler men into such a narrow space limit and when the price—$2.00—is taken into consideration the book seems a marvel of perfection and illustration.

---

American Edition of Nothnagel's Practice. Tuberculosis and Acute General Miliary Tuberculosis. By Dr. G. Cornet, of Berlin. Edited, with additions, by Walter B. James, M.D., Professor of the Practice of Medicine in the College of Physicians and Surgeons (Columbia University), New York. Octavo, 806 pages. Philadelphia, New York, London: W. B. Saunders & Company. 1904. (Cloth, $5.00 net; half morocco, $6.00 net.)

Not second to professional interest in tuberculosis is that of the people in general,—an awakening that has taken place within the last few years, or since the infectious nature of the disease has been determined. In devoting this volume to the consideration of tuberculosis the author and editors have shown wisdom.

This important disease receives the most elaborate handling in every aspect. Etiology takes up the entire first part, consisting of 345 pages, wherein every known factor contributing to the origin of the disease or playing any part in its causation is considered. Infection and methods of transmission are especially important just now; hence, as might be expected, these topics are exhaustively set forth.

The second part of the treatise deals with pulmonary tuberculosis. Early diagnosis is all important as every physician of intelligence knows, because it is in the early stages of the malady that curative treatment can be resorted to with confidence. Every useful method of diagnosis is described in detail, particular attention being given to examination of sputum, tuberculin, and serum diagnosis. The author and editor are committed to the use and value of tuberculin in the diagnosis of obscure cases, and of its harmlessness when properly used they are assured. The editor regards Koch's old tuberculin as the best.

Acute general miliary tuberculosis forms the concluding chapters of this most interesting book. The bibliography of tuberculosis is very extensive and is given here in 110 pages, even with abbreviated references, making the most valuable index of the literature of this disease that has yet been published. The entire volume constitutes a distinct addition to medical letters of the most advanced type, and will be appreciated by the scholars of the profession.

---

LEA'S SERIES OF MEDICAL EPITOMES. PEDIATRICS. A Manual for Students and Practitioners. By HENRY ENOS TULEY, A.B., M.D., Professor of Obstetrics in the Medical Department of Kentucky University, Louisville. Duodecimo, pp. 266, with 33 engravings. Series edited by V. C.Pedersen, M.D., Instructor in Surgery at the New York Polyclinic Medical School and Hospital. New York, Philadelphia: Lea Brothers & Co. 1903. (Price, $1.00.)

The importance of the study of diseases of children is recognised in most medical colleges, separate chairs having been assigned to this topic. In order to keep pace with the demands of undergraduate students, who ask for condensed literature on pediatrics, Professor Tuley has prepared this excellent epitomé. It contains the essentials of the subject as taught by the foremost instructors, authors, and specialists, and is a modern exposition of the best pediatric thought of teachers throughout the world. Infant feeding has been presented in accordance with its importance, leaving nothing to be added that could contribute to the value of such a book.

---

A MANUAL OF FEVER NURSING. By REYNOLD WEBB WILCOX, M.D., Professor of Medicine in the New York Post-Graduate Medical School and Hospital. Duodecimo, pp. 236. Philadelphia: P. Blakiston's Son & Co. 1904.

Special literature is increasing rapidly in every branch of medicine, and that pertaining to nursing has kept pace with the other topics in this regard. It has remained for Professor Wilcox, however, to present the subject of fever nursing and, it must be confessed, he has done the work most completely, not omitting any essential feature of this important special line of nursing. Perhaps there is no class of disease that demands of the nurse more skill, patience, and good judgment than fevers, especially typhoid, and every nurse who takes charge of such patients will appreciate this excellent manual. Physicians, likewise, would do well to read it, and to recommend it to their nurses.

A TEXTBOOK OF PHYSIOLOGY. By ISAAC OTT, A.M., M.D., Professor of Physiology in the Medico-Chirurgical College of Philadelphia. With 137 illustrations. Octavo, 563 pages. Philadelphia: F. A. Davis Company. 1904. (Price, $3.00 net.)

Physiology being one of the fundamentals of medicine, every opportunity to acquire it should be afforded the student. The laboratory is absolutely necessary, if one would become proficient, for the prosecution of this study and it should be presided over by a competent teacher. Next in importance are useful text-books, of which there are several at hand. This new candidate for favor is prepared by a competent teacher who yields to the solicitation of his pupils in presenting it to the profession. It is well arranged and contains the material needed in the laboratory; indeed, it may be called a guide to the laboratory study of physiology, though much of the laboratory technic has been omitted.

The author does not claim for it the dignity of a treatise, it being too elementary in character to justify that classification, but he considers it a work which contains the chief facts of physiology, indeed, all that are necessary for the student who would apply them in the practice of medicine. The illustrations are taken from various works that have been published heretofore, credit for which has been scrupulously given. The author does not lay claim to original work, but he has grouped the essential facts of physiology in accessible form.

---

MANUAL OF CLINICAL MICROSCOPY AND CHEMISTRY FOR STUDENTS AND PRACTITIONERS OF MEDICINE. By Dr. HERMANN LENHARTZ, Professor of Medicine and Director of Hospital at Hamburg, etc. Authorised translation from the fourth and last German edition, with notes and additions, by Henry T. Brooks, M.D., Professor of Histology and Pathology at the New York Post-Graduate Medical School and Hospital. With 148 illustrations and 9 colored plates. Pages xxxii-412. Octavo. Philadelphia: F. A. Davis Company. 1904. (Price, $3.00 net.)

The eminent author of this manual has prepared it with the sole object in view of supplying to students and physicians instruction in clinical microscopic and chemic methods of examination and, withal, to aid them in interpreting correctly the diagnostic significance of their findings. The microscopic portion of the book is limited, and very properly so, to the examination of fresh and dried impressions and teased preparations, thus not trenching upon the domain of pathology to which the examination of sections, and the like, belongs. The elements of microscopy, however, are carefully detailed, enabling the student to begin his work with the guidance of this book.

The chemical side of the manual is well conceived and equally well carried out. Vegetable and animal parasites are dealt with at length in the first section, bacteriology being set forth in ample form for the beginner, though the needs of the practitioner as well are conserved. The examination of the blood, sputum, gastric and intestinal contents, the urine and the aspirated fluids, each constitute separate sections which are handled with satisfaction and thoroughness.

It is proper to say something about the work of the translator. The difficult task of rendering such a work into smooth English has been done in a most admirable manner by Professor Brooks who, in addition, has inserted notes and illustrations which serve to bring the manual into nearer touch with the general practitioner and undergraduate student. These additions are inclosed in brackets, which make them distinctive and easily recognised. The translator dedicates his part of the work to Professor Daniel Bennett St. John Roosa, president of the New York Post-Graduate Medical School, a most befitting tribute to the attainments and labors of his chief.

It is proper to add that this is one of the really useful books for student and junior practitioner to study and consult; it is a distinct addition to the meritorious literature of the year and should receive the consideration at the hands of the profession that it richly deserves.

---

The Practical Medicine Series of Year Books. Ten volumes. Issued under the general editorial charge of Gustavus P. Head, M.D., Professor of Laryngology and Rhinology in the Chicago Post-Graduate Medical School. Volume IV. Gynecology. Edited by E. C. Dudley and William Healy, Chicago. Duodecimo, pp. 216. Chicago: The Year Book Publishers. 1904. (Price, $1.50; entire series, $5.50, payable in advance.)

The first part of this volume contains useful hints relating to technic that will prove of interest to all junior gynecologists. The position of the patient during gynecological examination and treatment, the use of the vaginal douche, the curet, anesthesia, ovarian grafting and other important matters are grouped under "general principles" in this section.

In other parts may be found articles relating to infectious and allied disorders; tumors and malformations; traumatisms; displacements, and disorders of menstruation and sterility. One of the editors, E. C. Dudley, has presented in detail the subject of secondary perineorrhaphy, with several illustrations, the object being to simplify and unify it, eliminate errors and accentuate important features of operative technic. No one is better qualified for this task than Dudley who is an expert in plastic gynecology, as well as in abdominal surgery.

In the section on displacements, Carstens's article, presented last year at Chicago before the American Association of Obstetricians and Gynecologists, wherein he advocated, in selected cases, the use of the stem pessary in the treatment of retroversion, is quoted at some length. Under the head of tumors, R. B. Hall's article on cystic kidneys resembling ovarian cysts, read at the same meeting, is abstracted. Among the other articles read at the meeting above referred to and which are dealt with under their appropriate heads, are J. B. Murphy's on tuberculosis of the female genitalia; R. T. Morris's on ovarian grafting; Joseph Prices's on death following pelvic and abdominal operations; E. J. Ill's on the Gilliam operation, and A. Goldspohn's on the technic of vaginal drainage.

The literature of the year has been well sorted and the best of it has been presented in this book, the editors having again displayed excellent judgment in performing a difficult task.

---

Diseases of the Intestines. A Textbook for Practitioners and Students of Medicine. By Max Einhorn, M.D., Professor of Medicine at the New York Post-Graduate Medical School and Hospital. Small octavo, pp. 397. Illustrated. Second, revised edition. New York: William Wood & Company. 1904. (Price, $3.00.)

Einhorn is not a new author nor is his work on diseases of the intestines his only book. His treatise on diseases of the stomach has already passed through three editions and another is preparing. The first edition of this work on the intestines issued from the press about four years ago, and has become exhausted. The demand for it, however, is such that a new edition becomes necessary, despite the fact that very little advance has been made in this field since the first appearance of Einhorn's treatise.

In the Journal for February, 1901, p. 543, we expressed a favorable impression of this textbook, and further observation and experience has served to confirm our previous judgment in the premises. We then pointed out the value of Einhorn's views on nervous affections of the intestines, and wish now to add a word in regard to his discussion of acute and chronic intestinal catarrh. The clinician is always glad of every serviceable hint in this troublesome condition which is here dealt with intelligently. Ulcers of the intestines command more attention since surgery, in appropriate cases, may be invoked with favorable prospects. The subject is one of interest and is interestingly treated in this book.

---

A Manual of Clinical Diagnosis by Means of Microscopical and Chemical Methods. For Students, Hospital Physicians and Practitioners. By Charles E. Simon, M.D., of Baltimore. Fifth edition, thoroughly revised and enlarged. Illustrated with 150 engravings and 22 plates in colors. Octavo, pp. 695. Philadelphia and New York: Lea Brothers & Co. 1904. (Price, $4.00.)

For the fifth time in the short period of eight years we are called upon to examine and report upon the qualities of this book. This is an indication of an increasing appreciation by students and practitioners of medicine, of the importance of a knowledge of the exacter methods of diagnosis which are distinguishing features of present day instruction. The literature of the past two years has been replete with new material relating to the blood, and here, in a chapter of nearly 200 pages it is found, it being

60 pages larger than the corresponding chapter in the preceding edition, embracing, too, an entirely new section on kryoscopic examination of the blood. Other sections, also, have been enlarged and still others material changes and additions have been made, until now the manual is as near complete as it is possible to make it.

We desire to invite special attention to the sections on the gastric juice and stomach contents, the feces, and the urine as being exhaustive in the treatment of these several subjects, clear in their presentation, and authoritative in method. Worthy of mention, too, are the sections on transudates and exudates, the semen, vaginal discharges, and the secretion of the mammary glands. Illustrations are made use of whenever necessary to amplify the text, some of the plates being superb. It is a book that no physician who practises modern medicine can deny himself without serious disadvantage.

---

The Medical News Pocket Formulary. By E. Quin Thornton, M.D., Assistant Professor of Materia Medica in the Jefferson Medical College, Philadelphia. New (sixth) edition. Leather, wallet shape for the pocket. Philadelphia and New York: Lea Brothers & Co. 1904. (Price, $1.50 net.)

This useful pocket reference book will be greeted with friendly words as it again appears, rejuvenated and brought forward to the present date. It claims many old friends as indicated by the demand for previous editions, and no doubt will gain many new ones through this new sixth republication. It contains hints for treatment as well as suggestions relating to incompatibles, poisons, antidotes, and other useful data for the sick room, either for emergencies or the routine course of practice.

---

Manual of Materia Medica and Pharmacy. Specially designed for the use of Practitioners and Medical, Pharmaceutical, Dental, and Veterinary Students. By E. Stanton Muir, Ph.G., V.M.D., Instructor in comparative Materia Medica and Pharmacy in the University of Pennsylvania. Third edition, revised and enlarged. Octavo, 192 pages. Philadelphia: F. A. Davis Company. 1904. (Price, $2.00 net.)

The first issue of this work was published about eight years ago, the second four or five years later, and now the third edition is presented for professional favor. Some changes have been made since the book first appeared, notably in eliminating many drugs, new and old, and retaining for description only those of recognised value in every day practice. The names of drugs are arranged in alphabetical order, which makes them easier for students to comprehend and quite as convenient for physicians to examine. The metric system is first given, and then, in parentheses, the equivalents in apothecaries' weights. A liberal interleaving in blank affords an opportunity for remarks and references. This is one of the most practical of manuals and is an excellent condensed exposition of materia medica and pharmacy.

Progressive Medicine. Volume II., June, 1904. A Quarterly Digest of Advances, Discoveries and Improvements in the Medical and Surgical Sciences. Edited by Hobart Amory Hare, M.D., Professor of Therapeutics and Materia Medica in the Jefferson Medical College of Philadelphia. Philadelphia and New York: Lea Brothers & Company. (Per annum, in four cloth-bound volumes, $9.00; in paper binding, $6.00, carriage paid to any address.)

This number contains articles on surgery of the abdomen, including hernia, by William B. Coley; gynecology, by John G. Clark; diseases of the blood, diathetic and metabolic diseases, and diseases of the spleen, thyroid gland and lymphatic system, by Alfred Stengel, and ophthalmology, by Edward Jackson.

It may be that some prefer this book unbound, but frankly we do not, an unbound volume being practically of no use in our library.

